# Four Derivative Spectrophotometric Methods for the Simultaneous Determination of Carmoisine and Ponceau 4R in Drinks and Comparison with High Performance Liquid Chromatography

**DOI:** 10.1155/2014/650465

**Published:** 2014-02-12

**Authors:** Fatma Turak, Mithat Dinç, Öznur Dülger, Mahmure Ustun Özgür

**Affiliations:** ^1^Department of Chemistry, Faculty of Science and Art, Yıldız Technical University, 34220 Istanbul, Turkey; ^2^Tekirdag City Food Control Laboratory, 59000 Tekirdag, Turkey

## Abstract

Four simple, rapid, and accurate spectrophotometric methods were developed for the simultaneous determination of two food colorants, Carmoisine (E122) and Ponceau 4R (E124), in their binary mixtures and soft drinks. The first method is based on recording the first derivative curves and determining each component using the zero-crossing technique. The second method uses the first derivative of ratio spectra. The ratio spectra are obtained by dividing the absorption spectra of the binary mixture by that of one of the components. The third method, derivative differential procedure, is based on the measurement of difference absorptivities derivatized in first order of solution of drink samples in 0,1 N NaOH relative to that of an equimolar solution in 0,1 N HCl at wavelengths of 366 and 451 nm for Carmoisine and Ponceau 4R, respectively. The last method, based on the compensation method is presented for derivative spectrophotometric determination of E122 and E124 mixtures with overlapping spectra. By using ratios of the derivative maxima, the exact compensation of either component in the mixture can be achieved, followed by its determination. These proposed methods have been successfully applied to the binary mixtures and soft drinks and the results were statistically compared with the reference HPLC method (NMKL 130).

## 1. Introduction

Color is a vital constituent of food and probably the first characteristic perceived by the human senses. Probably, for economic reasons, brightly colored and stable synthetic colorants have been widely used as food additives. These colorants are used to supplement and enhance natural colors destroyed during processing or storage and substantially increase the appeal and acceptability of food stuff. The term color additive can be applied to any dye, pigment, or other substances artificially made or obtained from a vegetable, animal, mineral, or another natural source [1]. Some synthetic colorants and their metabolic products are consumed in large amounts maybe toxic and cause cancer, deformation, and so forth [2].

**Scheme 1:**
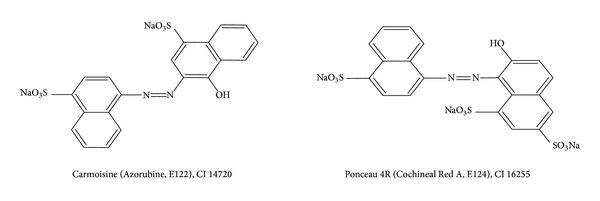
Chemical structures, common names, European community numbers (E), and color index (CI) numbers of synthetic food colorants studied.

Carmoisine (E122) and Ponceau 4R (E124) are synthetic dyes that contain azo and aromatic ring structures and are often used in dyeing food, drink, medicine, and cosmetics (Scheme 1).

The development of chemical and instrumental methods for the separation, identification, and quantitative analysis of synthetic food colorants has become extremely important for the food and beverages industry and academic and governmental institutions to assess the quality and safety of food products [3]. Although the amounts of synthetic colors that are added to food and drinks are strictly controlled, they may exceed the authorized levels. Thus monitoring of the levels of colorants in high consumption products such as beverages becomes of great importance. The number of works published during the last years demonstrates the importance of this problem and the need for developing fast, accurate, and selective techniques for synthetic colorants analysis [4].

Various techniques have been introduced for the determination of mixtures of colorants with three or more component combination in food samples.

These include capillary electrophoresis [5], differential pulse polarography [4], high performance liquid chromatography [6–9], electrospray mass spectrometry [10], spectrophotometry [11–13], partial least squares [14], and spectrofluorimetry [15]. Chromatographic methods have been used for colorant analysis in foods, and they are recommended when the mixture contains many different colorants [6, 7, 16, 17]. However, there are some potential drawbacks, such as usage of toxic solvents, the need for complex sample pretreatments, and the resulting waste product. Capillary electrophoresis is also very suitable when the sample contains several colorants but foods, beverages, and pharmaceuticals contain only one, two, or rarely three food dyes and these methods need special equipment that may not be available in certain quality laboratories. All multivariate calibration methods, including Partial Least Square (PLS), require the data processing with powerful software as well as the manipulations of the abstract vector space and its application to the regression analysis and various chemometric methods are also often used for more complex mixtures. Thus, analytical methods from alternative technique are always useful, especially if the methods are simple, cheap, and comparatively fast. Spectrophotometry, as alternative methodology, is suitable for routine laboratories especially for developing countries, and sometimes, serious analytical problems can be resolved by this common technique. Among computer-controlled instruments, derivative techniques (ratio derivative, difference derivative, and compensation method) are playing a very important role in the binary analysis of mixtures by UV-VIS molecular absorption spectrophotometry. All approaches are useful in the resolution of band overlapping in quantitative analysis.

In this present work, selective, sensitive, simple, and low-cost procedures were developed for the simultaneous determination of Carmoisine (C) and Ponceau 4R (P) in synthetic mixture and drink samples by using derivative spectrophotometry (DSM), ratio derivative spectrophotometry (RDM), derivative differential spectrophotometry (DDM), and compensation method (CM). These methods have been successfully applied to synthetic mixtures and drink samples and the results obtained were compared with those obtained by HPLC method according to NMKL 130 [18].

No extraction or evaporation step, no complexation agent, and no harmful chemicals are involved in the suggested methods, in the suggested methods, so can be used for routine analysis of both colorants in quality control and routine laboratories.

## 2. Experimental

### 2.1. Apparatus

All spectral measurements and treatment of data were carried out using a Shimadzu UV-2450; double beam UV-Vis spectrophotometer with two matched 1-cm quartz cells, connected to a Hewlett Packard compatible personal computer and a HP 1102 Laser Jet Printer. Bundled UV-PC personal spectroscopy software of version 2.21 was used to process the absorption and the derivative spectra. The spectral band width was 1 nm with wavelength scanning speed of medium. HPLC measurements were performed with an Agilent 1100 series HPLC-DAD system with a vacuum degasser, quaternary pump, auto sampler, and injector with a 100 *μ*L loop volume. Separation was achieved using a Waters Spherisorb C18 column (4.6 × 250 mm, 5 *μ*m) and variable wavelength UV-VIS DAD detector measuring at 520 nm for Carmoisine and 512 nm for Ponceau 4R.

### 2.2. Chromatographic Conditions

Separation was achieved using a Waters Spherisorb C_18_ column, (4.6 × 250 mm, 5 *μ*m). The solvent system consisted of a mixture of an aqueous solution containing 0.005 M tetrabutylammonium hydrogen sulphate (TBA) and a phosphate buffer solution (pH 6.5): methanol (50 : 50 v/v). It was pumped isocratically at 0.7 mL min^−1^ for the separation. The eluents were monitored at 512 and 520 nm for P and C, respectively.

### 2.3. Chemicals and Materials

Distilled water was used to prepare the solutions and for mobile phase. All chemicals used were of analytical reagent grade and were purchased from Merck (Darmstadt, Germany). Ponceau 4R (E124, Cochineal Red A, molecular weight 604.47, CI 16255 CAS number 2611-82-7) and Carmoisine (E122 Azorubine, molecular weight 502.44, CI 14720,CAS number 53026-69-9) were from Sigma-Aldrich (Steinheim, Germany). Soft drinks were purchased from commercial markets.

### 2.4. Standard Solutions and Calibration Graphs

Stock solutions of Carmoisine and Ponceau 4R (100 *μ*g mL^−1^) were prepared by dissolving appropriate weights in distilled water. Various aliquots of each stock solution, within the concentration range stated in Table 2, were transferred into five sets of 10 mL volumetric flasks and the volume was completed with distilled water. The stability of the working solutions of Carmoisine and Ponceau 4R was studied by recording the time-dependent absorption spectra; no changes in the spectra were observed for at least one month when the solutions are stored at room temperature in the dark.

For DDM method, accurate volume aliquots of the stock solution were transferred into two sets of 10 mL volumetric flasks and the volume was made up with 0.1 N HCI and 0.1 N NaOH to give a series of equimolar solutions containing 2.0–10.0 *μ*g mL^−1^ of both Carmoisine and Ponceau 4R. Calibration solutions (*n* = 5) were used to construct the calibration curves in the standardization of the cited methods.

For HPLC method, stock solutions of (25 mg/100 mL) Carmoisine and Ponceau 4R were prepared in a 1 : 1% v/v mixture of methanol—TBA buffer solution (0.005 M TBA + 0.005 M phosphate buffer, pH 6.5). The standard solutions were prepared containing 5–20 *μ*g mL^−1^ of Carmoisine and Ponceau 4R in the same solvent system. All prepared solutions were filtered through 0.45 *μ*m membrane filter before injection.

Commercial drink samples due to turbid are treatment with the Carrez I and II solutions.

 Carrez solution I: it contains potassium hexacyanoferrate (II) trihydrate (K_4_[Fe (CN)_6_] × 3H_2_O) 100 mL concentration: 15 g × 100 mL^−1^
 Carrez solution II: it contains zinc sulfate heptahydrate (ZnSO_4_  × 7H_2_O) 100 mL concentration: 30 g × 100 mL^−1^.

### 2.5. Spectrophotometric Measurements

- Final solutions for measurements were prepared over the concentration ranges given in Table 2.
- The derivative spectra were recorded over the wavelength range of 300–700 nm. The amplitude of the derivative curve was measured at the selected wavelengths for each method (Table 1).
- For the compensation method the absorbance difference spectra (sample versus reference) were recorded over the wavelength range of 300–700 nm, using different concentrations of the reference solution, prepared from the stock solution.
- For the DD method, the Carmoisine and Ponceau 4R solutions in 0.1 N HCI difference derivative spectra were recorded against corresponding 0.1 N NaOH solutions of the colorants as a blank. The amplitude of the first derivative spectra (Table 2) was measured at the chosen wavelengths.

### 2.6. Procedures

#### 2.6.1. Derivative Spectrophotometric Method (DSM)

Aliquots of Carmoisine working solutions equivalent to 2.0–10.0 *μ*g mL^−1^ were placed into 10 mL calibration flasks and the volume was made up with distilled water. The absorption spectra were recorded versus distilled water. Next the zero-order spectra were transformed into first derivative. The amplitude from the baseline to peak at 331 nm (zero-crossing point for Ponceau 4R) was related to the actual contents of Carmoisine.

Appropriate aliquots of aqueous working solutions of Ponceau 4R were placed into 10 mL calibration flasks and the volume was made up with distilled water. The spectra of standard solutions in the concentration range 2.0–10 *μ*g mL^−1^ were recorded and subjected to the mathematical treatment (first derivative). The values of ^1^D_517_ measured at 517 nm (zero-crossing point for Carmoisine) were used for construction of calibration graph.

#### 2.6.2. Ratio Derivative Spectrophotometric Method (RDM)

Accurately aliquots of 0.2, 0.4, 0.6, 0.8, and 1.0 mL of colorants stock solution were transferred into 10 mL volumetric flasks and the volume was made up with distilled water. The spectra of the prepared standard solutions were scanned from 300 to 700 nm and stored in the computer. For the determination of Carmoisine, the stored spectra of Carmoisine were divided separately by the spectrum of 6 *μ*g mL^−1^ Ponceau 4R to obtain the ratio spectra. The first derivative corresponding to each ratio spectrum (^1^DD) was recorded. A calibration graph relating the peak amplitudes (^1^DD_323_) to the corresponding concentrations in *μ*g mL^−1^ of Carmoisine was constructed.

In order to determine Ponceau 4R, stored spectra of Ponceau 4R standard solutions were divided by a standard spectrum of Carmoisine of 6 *μ*g mL^−1^. The first ratio derivative spectra were calculated. The concentration of the Ponceau 4R was proportional to the amplitude of the maximum at 344 nm (^1^D_344_).

#### 2.6.3. Derivative Differential Spectrophotometric Method (DDM)

The difference spectra between the acidic (0.1 N HCI) solution and equimolar basic (0.1 N NaOH) solution of pure colorants and drink samples were recorded from 300.0 to 700.0 nm by placing the acidic solution in the sample compartment and the basic solution in the reference compartment. A first derivative spectrum of each of the differential curves was subsequently recorded. The derivative absorbance values were measured at 366 and 451 nm for Carmoisine and Ponceau 4R, respectively.

#### 2.6.4. Compensation Method (CM)

*(1) Determination of Standard Ratios*. The first derivative spectra for each set of standard solutions were recorded using the appropriate blank solution. The ratios of the first derivative maxima and minima (^1^D_*λ*_1__/^1^D_*λ*_2__), where appropriate at the specified wavelengths (*λ*_1_ and *λ*_2_) as indicated in Table 1, were calculated.

*(2) Compensation Spectrophotometric Measurements*. The solution of the mixture (containing compounds Carmoisine and Ponceau 4R) was placed in the sample cell. A series of solutions containing various concentrations (2–10 *μ*g mL^−1^) of Carmoisine above and below that present in the mixture solution were prepared and placed in succession in the reference cell. The first derivative spectra (^1^D) were recorded and the corresponding ratios (Table 1) in each instance were calculated and followed the calculated ratio for pure colorant Ponceau 4R. The exact balance point (the ratio of the mixture is equal to that of pure compound Ponceau 4R) was determined at which the concentration of Carmoisine in sample solution is equal to that in the reference solution.

By analogy, the same steps were followed by using solutions of pure Ponceau 4R in the reference cell to determine its concentration in the mixture at the balance point.

The same principle was also applied for the analysis of drink samples.

*(3) Graphical Method*. To avoid the excessive preparation of several standard solutions of either compound Carmoisine or in order to locate the exact balance point, a graphical method is recommended [19, 20].

The same steps as for the compensation method were followed and the derivative ratio of the mixture (in the sample cell) was calculated at each time and plotted against the concentration of pure compound Carmoisine (in the reference cell). A line with slight curvature is usually obtained. The concentration of Carmosine can easily be interpolated from the graph by substituting the ratio of pure Ponceau 4R. At the point at which the ratio of the mixture is equal to that of pure Ponceau 4R, the concentration of Carmoisine in the reference cell is equal to that in the mixture in the sample cell. By analogy, the concentration of compound Ponceau 4R can be obtained.

#### 2.6.5. HPLC Method (Reference Method; NMKL 130)

This reference method is described in detail in Nordic Committee on Food Analysis [18]. About 20 *μ*L of each sample was injected and the concentrations were calculated on the basis of the ratios of peak areas with those of the standard solutions. The chromatograms of two compounds and drink samples were plotted and stored in the computer. Separation was carried out in room temperature. The mobile phase was prepared daily, filtered through a 0.45 *μ*m membrane filter. All chromatograms were run with the use of a (50 : 50, v/v) mixture of methanol and TBA buffer. Other experimental parameters were flow rate, 0.7 mL min^−1^, retention times, 5.54 min for Ponceau 4R, and 21.62 min for Carmoisine.

#### 2.6.6. Application of the Proposed Methods for the Determination of Two Colorants in Drink Sample

Commercial samples were mixed with Carrez (I) and Carrez (II) solutions. Once precipitated, the samples were centrifuged. 50 mL of the sample that is clarified by Carrez precipitation was transferred to 100 mL volumetric flask and the volume was made up with distilled water. A series of dilution was prepared quantitatively with water from this solution to obtain standard solutions to reach the concentration ranges of calibration curves graphed for each of the proposed methods (DSM, RDM, and CM). For the DDM method, the same procedure was used, but in this case the final volume was made up with 0.1 N HCI and 0.1 N NaOH, separately for the determination of Carmoisine and Ponceau 4R. HPLC method, the same sample preparation procedure described above, was followed using a mixture 1 : 1, v/v of methanol—TBA buffer solution. After dissolution process, prepared solutions were filtered using 0.45 *μ*m disposable membrane filters (Sartrious, Minisart, 0.45 *μ*m) by using a syringe. The final solution was diluted to the working concentration range.

### 2.7. Method Validation

The validity and suitability of the proposed methods were assessed by accuracy (reported as percentage recovered), precision (reported as RSD%), linearity (evaluated by regression equation), limit of detection (LOD), and limit of quantification (LOQ) [21].

LOD and LOQ were based on the standard deviation of response and the slope of the corresponding curve using the following equations:

(1) $$\begin{array}{c} \text{LOD}=3\frac{s}{m};\;\;\text{LOQ}=10\frac{s}{m}, \end{array}$$

where *s*, the noise estimate, is the standard deviation of the derivative amplitude of the blank and *m* is the slope of the related calibration graphs [22].

The linearity of the methods was established in the concentration ranges of 2–10 *μ*g mL^−1^ for Carmoisine and Ponceau 4R, respectively. In this study five concentrations were analyzed in five replicates. The linearity was evaluated by the least square regression method.

In order to demonstrate the validity and suitability of the proposed methods, intra- and interday variability studies were performed at three different concentrations (2, 6, and 10 *μ*g mL^−1^), in the presence of the certain concentration (2, 6, and 10 *μ*g mL^−1^) of the other colorants, three times in a day and also three different days. Method efficiency was tested in terms of RSD's for both intraday and interday precisions.

The precision was ascertained by carrying out five replicate determinations of synthetic mixtures at different concentration ratios of Carmoisine and Ponceau 4R. Recovery test also confirmed the accuracy and applicability of the proposed methods by analyzing several synthetic mixtures of Carmoisine and Ponceau 4R which reproduced different composition ratios within the linearity range. These ratios are also the ratios in drinks. The mean percentage recoveries and RSD values were calculated.

Recovery of the colorants of interest from a given matrix can be used as a measure of the accuracy or the bias of the method. The same ranges of concentrations as employed in the linearity studies were used.

In our study, the applicability of the derivative spectrophotometric methods was also validated by comparison with the HPLC technique.

## 3. Result and Discussion

**Figure 1:**
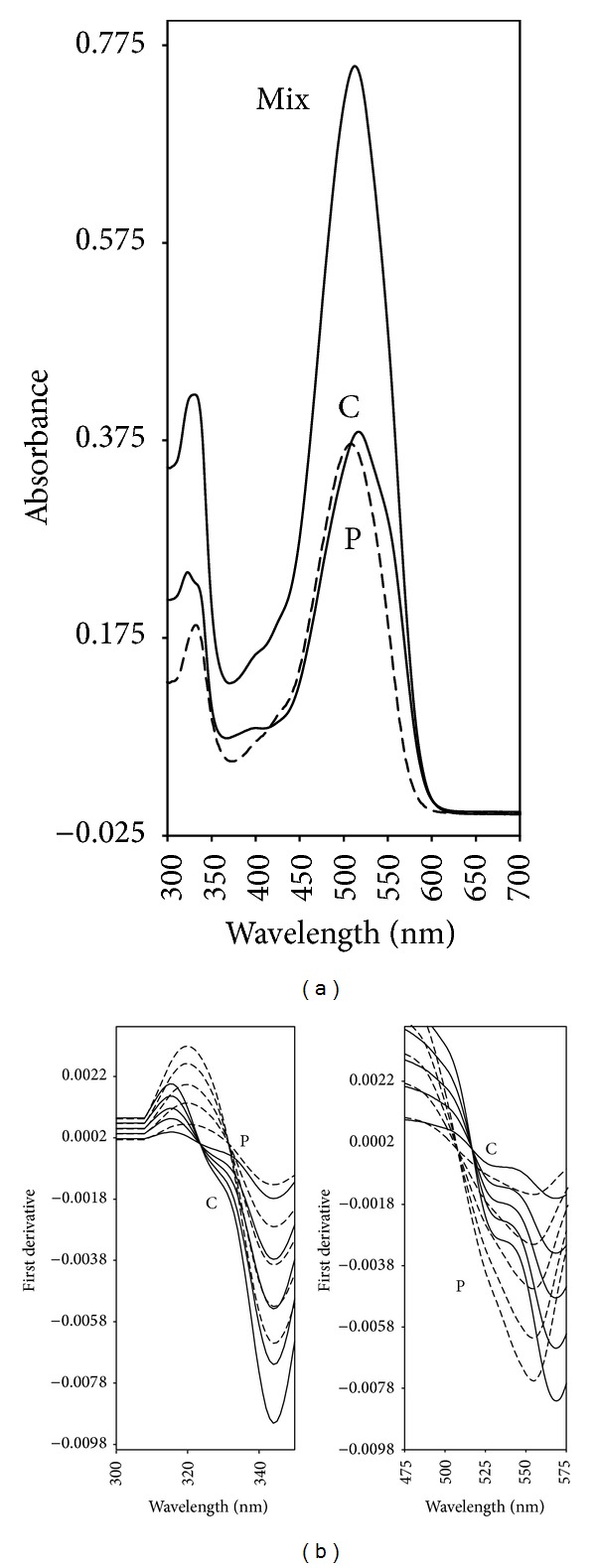
Zero-order spectra (a) of 10 *μ*g mL^−1^ Carmoisine, 10 *μ*g mL^−1^ Ponceau 4R, and their binary mixture; first derivative spectra (b) of colorants (Carmoisine(—): 2–10 *μ*g mL^−1^; Ponceau 4R (- -): 2–10 *μ*g mL^−1^).

Figure 1(a) shows the absorption spectra of Carmoisine, Ponceau 4R, and the mixture of them in the wavelength range of 300–700 nm. Zero-order absorption spectra of Carmoisine and Ponceau 4R in distilled water gave rise to maximum peaks at 517 nm and 508 nm, respectively. As can be seen, the spectra of the colorants were strongly overlapped in the 400–600 nm wavelength region. Hence, the direct absorption measurement for assaying binary mixture seems to be impossible. On the other hand, the absorption spectra of the two components within a wavelength range of 300–380 nm were less overlapped than the region 400–600 nm. So, in this work, the region of 300–380 nm was chosen for the analysis of Carmoisine. In this spectral range, DSM, RDM, and DDM were used for the determination of the studied colorants in laboratory prepared mixtures and soft drink samples. These methods are also applied to analyze Ponceau 4R in the region 320–520 nm.

### 3.1. Derivative Spectrophotometric Method (DSM)

Under computer-controlled instrumentation, derivative spectrophotometry played a very important role in the resolution of band overlapping in quantitative analysis [23].  Figure 1(b) shows the first derivative spectra of Carmoisine and Ponceau 4R. As can be seen in Figure 1(b), in the region of 300–520 nm, the first derivative spectra allowed the simultaneous determination of Carmoisine and Ponceau 4R. At this region, the ^1^D spectra (Figure 1(b)) show a characteristic peak at 331 nm for Carmoisine, while Ponceau 4R shows no response. Therefore, the absolute values of the ^1^D peak amplitudes at 331 nm can be used to quantify Carmoisine in this mixture. On the other hand, Ponceau 4R can be assayed in the presence of Carmoisine by measuring its ^1^D response at 517 nm (zero-crossing point of Carmoisine).

Under the experimental conditions described, the calibration graphs obtained by plotting the derivative values versus concentrations for Carmoisine and Ponceau 4R, in the concentration range stated in Table 2, exhibited linear relationships. Statistical analysis of the data showed that linearity of the calibration graphs and compliance with Beer's law were validated and the small values of the intercepts as illustrated by the excellent values of correlation coefficients of the regression equations. To prove the validity and applicability of the proposed method, laboratory-prepared mixtures were prepared at different concentration ratios of Carmoisine and Ponceau 4R (Table 3). The obtained results for the analysis of these mixtures, in five replicates by the described method, showed high accuracy and precision of the proposed method (Table 3).

### 3.2. Ratio Derivative Method (RDM)

While the main disadvantages of the zero-crossing method in derivative spectrophotometry for resolving a mixture of components with overlapped spectra are the risk of small drifts of the working wavelengths and the circumstance that the working wavelengths generally do not fall in correspondence of peaks of the derivative spectrum, this may be particularly dangerous when the slope of the spectrum is very high with consequent loss of accuracy and precision, and the working wavelength is in proximity of the base of the spectrum which causes poor sensitivity. The main advantage of the RDM is the chance of doing easy measurements in correspondence of peaks so it permits the use of the wavelength of highest value of analytical signals (a maximum or a minimum), and moreover, the presence of a lot of maxima and minima is another advantage by the fact that these wavelengths give an opportunity for the determination of active compounds in the presence of other compounds and ingredients which possibly interfere in the assay [24–27].

**Figure 2:**
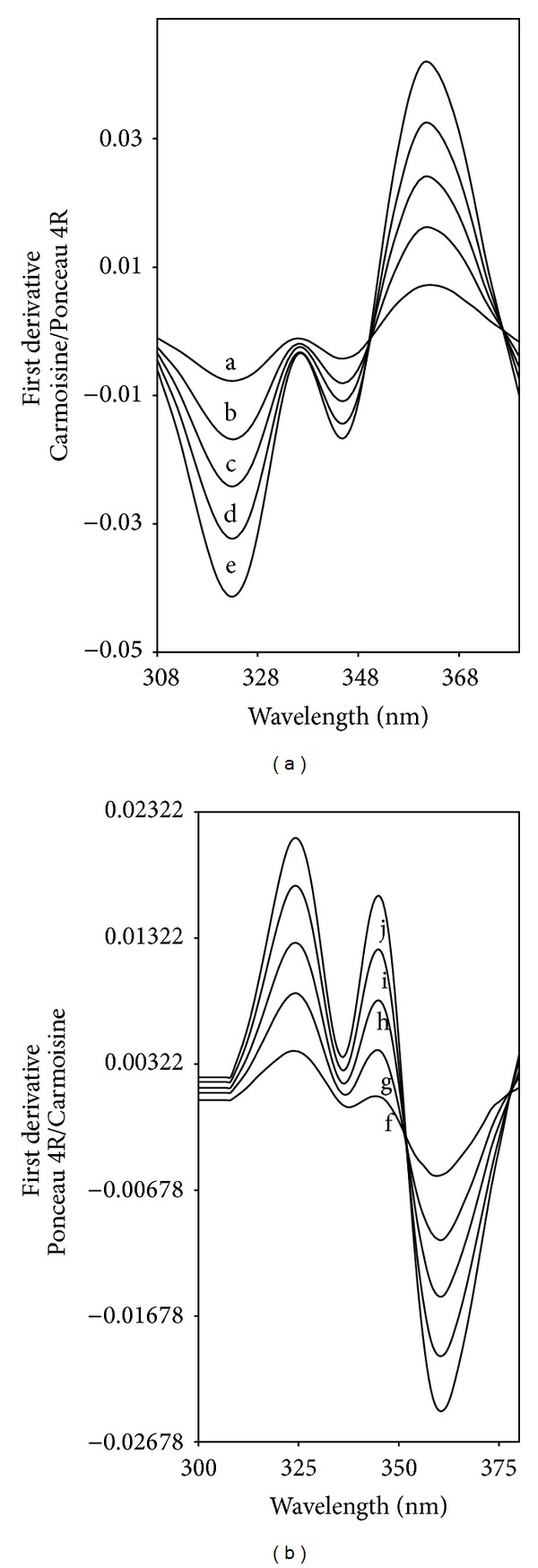
First derivative ratio spectra of Carmoisine (a) and Ponceau 4R (b) for different concentrations (Carmoisine (6 *μ*g mL^−1^ Ponceau 4R was used as a divisor); a: 2 *μ*g mL^−1^, b: 4 *μ*g mL^−1^, c: 6 *μ*g mL^−1^, d: 8 *μ*g mL^−1^, e: 10 *μ*g mL^−1^; Ponceau 4R (6 *μ*g mL^−1^ Carmoisine was used as a divisor); f: 2 *μ*g mL^−1^, g: 4 *μ*g mL^−1^, h: 6 *μ*g mL^−1^, i: 8 *μ*g mL^−1^, j: 10 *μ*g mL^−1^).

This method is based on the use of the first derivative of the ratio spectra. The ratio spectra were obtained by dividing the absorption spectrum of the mixture by that of one of the components. As we depicted above, direct absorption measurements are not possible due to spectral overlap. This spectral overlapping was sufficiently enough to demonstrate the resolving power of the proposed method.  Figure 2(a) shows the first ratio derivative spectra of different Carmoisine standard solutions (spectra divided by the spectrum of 6 *μ*g mL^−1^ Ponceau 4R solution). The ratio first derivative amplitudes at 323 nm (^1^DD_323_) corresponding to a maximum wavelength, are proportional to the Carmoisine concentration (Figure 2(a)). To determine the other component Ponceau 4R, an analogues procedure was followed. For determination of Ponceau 4R, the stored spectra of the mixtures are divided by a standard spectrum of 6 mg L^−1^ Carmoisine (Figure 2(b)). Standard solutions of Ponceau 4R and the content of Ponceau 4R were determined by measuring the signals at 344 nm (^1^DD_344_), Figure 2(b).

Under the described experimental conditions, the calibration graphs obtained by plotting the derivative values of each colorant versus concentration in the concentration range stated in Table 2 showed linear relationships. Conformity with Beer's law was evident in the concentration range of the final dilution cited in Table 2. For this method, the characteristic parameters of regression equations and correlation coefficients are given in Table 2. The correlation coefficients were 0.9996 and 0.9994 indicating good linearity. LOD and LOQ were calculated for each colorant and are shown in the same table. The detection limits were found to be 0.079 *μ*g mL^−1^ for Carmoisine and 0.082 *μ*g mL^−1^ for Ponceau 4R, while the quantification limits were estimated to be 0.263 *μ*g mL^−1^ for Carmoisine, and 0.273 *μ*g mL^−1^ for Ponceau 4R. The proposed method was also applied to the drink samples (Table 4). The accuracy of RDM was checked by analyzing the laboratory prepared mixtures of Carmoisine and Ponceau 4R at various concentration ratios ranging from 2 to 10 *μ*g mL^−1^ (Table 3). All data represent the average of five determinations. Low values of relative standard deviation indicate very good precision. Satisfactory recoveries with small standard deviations were obtained, which indicated the high repeatability and accuracy of the method.

### 3.3. Derivative Differential Method (DDM)

Differential spectrophotometry (ΔD_1_) based on pH changes has also been reported to be useful in the determination of binary mixtures. This method that depends on utilization of difference absorption spectra corresponding to the same compound obtained at two different pH's has been investigated for the analysis of binary mixtures. The procedure is comprised of the measurement of ΔD_1_ food colorants' in acidic solutions against their alkaline solutions as blanks. The ΔD_1_ has been successfully used to eliminate interferences from drink samples. There are few reports on utilization of the above two combined techniques (difference and derivative) for the estimation of individual drug substances and for combined preparations [28–30].

**Figure 3:**
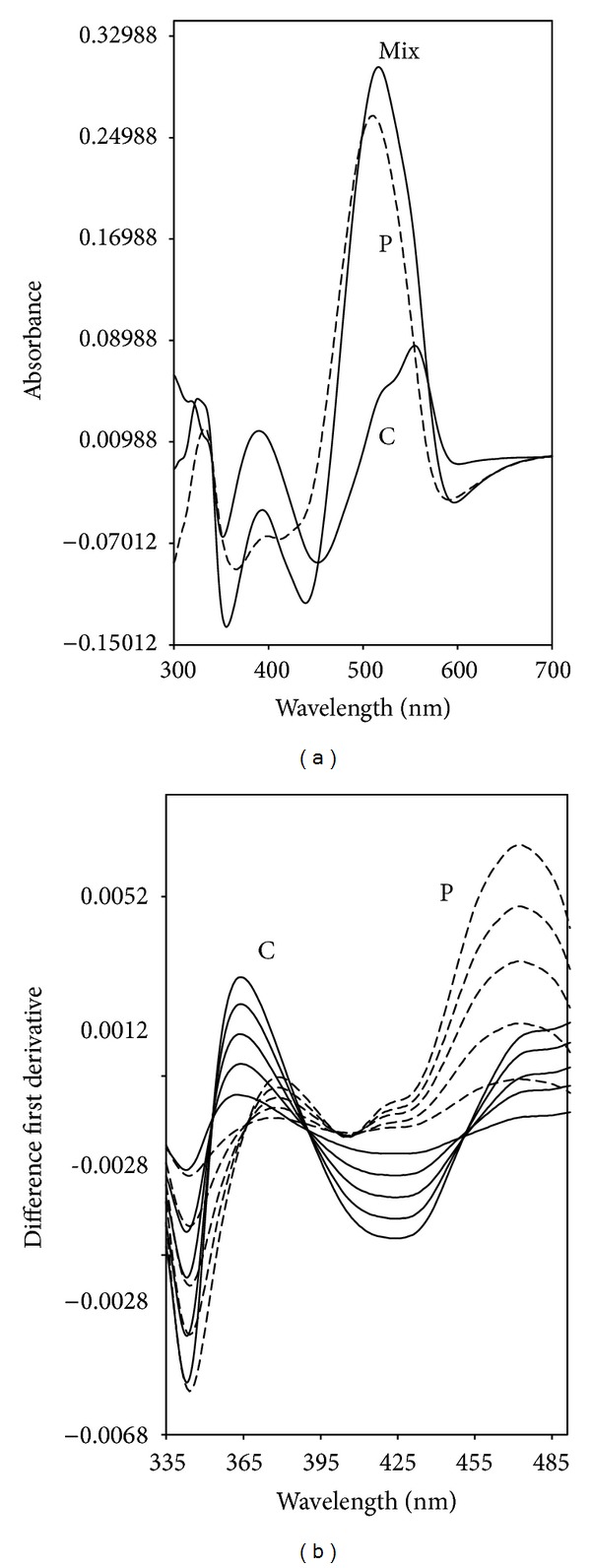
Difference absorption spectra of 10 *μ*g mL^−1^ Carmoisine, 10 *μ*g mL^−1^ Ponceau 4R and their binary mixture (a). Difference first derivative spectra (b) of colorants (Carmoisine: 2–10 *μ*g mL^−1^; Ponceau 4R: 2–10 *μ*g mL^−1^).

The difference absorption spectra of Carmoisine, Ponceau 4R, and their binary mixture in 0.1 N NaOH and in 0.1 N HCI are shown in Figure 3(a).  Figure 3(b) shows the first derivative difference spectra of colorants in the concentration range 2–10 *μ*g mL^−1^. The spectra of both colorants in Figure 3(b) offer an advantage for their simultaneous determination by having zero-crossing points. In particular the first derivative amplitudes at 366 nm for Carmoisine and 451 nm for Ponceau 4R in the Carmoisine and Ponceau 4R mixture were considered as the optimum working wavelengths for their determination. By measuring the values of the ΔD_1_ amplitudes at these wavelengths, the concentration of each colorant can be directly calculated since the differential first derivative measurement cancels the irrelevant absorbance due to the drinks matrix at these wavelengths.

Under the experimental conditions described, the calibration graphs obtained by plotting the derivative values of each colorant in the mixture versus concentration, in the range concentration stated in Table 2, show linear relationships. The correlation coefficients were 0.9996 and 0.9998 indicating good linearity. The LOD were 0.071 *μ*g mL^−1^ for Carmoisine and 0.081 *μ*g mL^−1^ for Ponceau 4R, while the LOQ were 0.236 *μ*g mL^−1^ for Carmoisine and 0.270 *μ*g mL^−1^ for Ponceau 4R. The proposed procedure was successfully applied for the determination of these colorants in laboratory-prepared mixtures and drink samples. The results are presented in Tables 3 and 4. The values of relative standard deviation indicate very good reproducibility. In addition, application of the DDM was found to be correct for the drink matrix interference and to enhance the sensitivity.

### 3.4. Compensation Method (CM)

**Figure 4:**
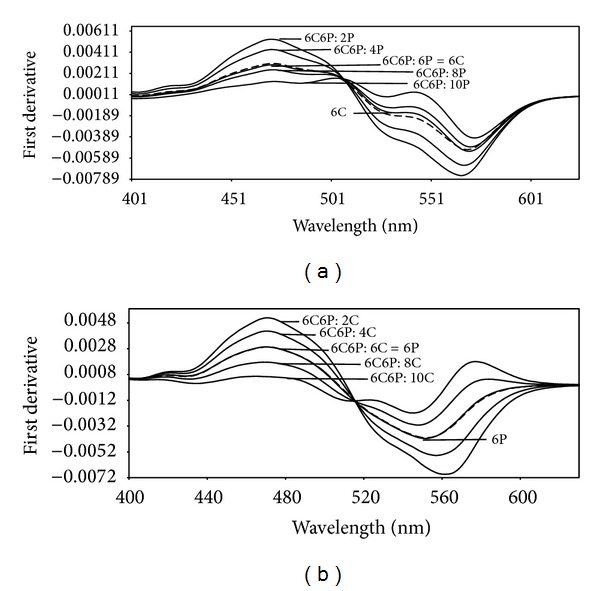
(a) First derivative spectra between the mixture solution (6 *μ*g mL^−1^ Carmoisine + 6 *μ*g mL^−1^ Ponceau 4R) in the sample cell and different concentrations (2–10 *μ*g mL^−1^) of Ponceau 4R solutions in the reference cell. First derivative spectra between the mixture solution (6 *μ*g mL^−1^ Carmoisine + 6 *μ*g mL^−1^ Ponceau 4R) in the sample cell and 6 *μ*g mL^−1^ Ponceau 4R in the reference cell at balance points and comparison of these spectra with 6 *μ*g mL^−1^ concentration of Carmoisine (- - -) spectrum. (b) First derivative spectra between the mixture solution (6 *μ*g mL^−1^ Carmoisine + 6 *μ*g mL^−1^ Ponceau 4R) in the sample cell and different concentrations (2–10 *μ*g mL^−1^) of Carmoisine solutions in the reference cell. Derivative spectra between the mixture solution (6 *μ*g mL^−1^ Carmoisine + 6 *μ*g mL^−1^ Ponceau 4R) in the sample cell and 6 *μ*g mL^−1^ Carmoisine in the reference cell at balance points and comparison of these spectra with 6 *μ*g mL^−1^ concentration of Ponceau 4R (- - -) spectrum.

The compensation method is a nonmathematical method for the detection and elimination of unwanted absorption during spectrophotometric analysis [19, 20, 31, 32]. The accuracy of the method depends on the evaluation of the balance point.  Figure 1(b) shows the first derivative spectra of Carmoisine and Ponceau 4R in the concentration range 2–10 *μ*g mL^−1^. The first derivative spectra were recorded for each reference solution of the components and the ratios of the ^1^D maximum and ^1^D minimum were calculated. Table 1 shows the mean values of the ratios calculated for five different determinations for each solution. The ratios are constant and characteristic of the pure substance, independent of concentration and presence of another component. For the determination of Carmoisine concentrations in Carmoisine-Ponceau 4R binary mixtures and drink samples, the sample cell was filled with the mixture solution (6 *μ*g mL^−1^ Carmoisine + 6 *μ*g mL^−1^ Ponceau 4R) and the reference cell was filled, in succession, with a series of reference Ponceau 4R with solutions in different concentrations Ponceau 4R, as shown in Figure 4(a). The ratios of the mixture calculated from the recorded ^1^D spectra were compared with those of Carmoisine. At the exact balance point, the ratio of the mixture corresponds to that of Carmoisine where the concentration of Carmoisine in the mixtures in the sample cell is equal to that of the reference solution in the reference cell. For determining the other component, the same steps were followed using solutions of pure compound Carmoisine in the reference cell to determine its concentration in the mixture (Figure 4(b)). Conformity with Beer's law was evident in the concentration range from 2 to 10 *μ*g mL^−1^ of Carmoisine and Ponceau 4R. In this method, synthetic mixtures were prepared at different concentration ratios of Carmoisine and Ponceau 4R and the recoveries of the method were found to be in the range of 98.50 ± 1.05%–99.75 ± 1.10%. The presented results in Table 3 are in good agreement with the other proposed methods.

### 3.5. Application of the Developed Methods to the Soft Drinks

The developed and validated methods were successfully applied to the determination of Carmoisine and Ponceau 4R content in the commercial soft drinks. The obtained results are presented in Table 4. Good agreement was observed for the assay results of the drinks by application of the four methods in this paper (Table 4). The values of standard deviation indicate very good reproducibility. The results of the proposed spectrophotometric methods were statistically compared with the results of the HPLC method given in the literature [18] at the 95% confidence level with student's *t*-test and by the variance ratio *F*-test (Table 4). The calculated *t*- and *F*-values never exceed the theoretical *t*- and *F*-values at 0.05 level of significant difference. There was no interference of ingredients in the products examined, so no additive extraction or separation procedures were required during their assay. The results of all methods were very close to each other. This suggests that the four methods are equally applicable.

### 3.6. Validation of the Proposed Methods

#### 3.6.1. Linearity

The linearity of the proposed methods was evaluated under different concentrations of Carmoisine and Ponceau 4R within the concentration range stated in Table 2. The good linearity of the calibration graphs and negligible scatter of the experimental points is clearly evident by the values of the correlation coefficients and variances around the slope (Table 2).

#### 3.6.2. Limit of Detection (LOD) and Limit of Quantification (LOQ)

LOD is expressed as the analyte concentration corresponding to the sample blank value plus three standard deviations and LOQ is the analyte concentration corresponding to the sample blank value ten standard deviations [21]. All the proposed methods data was presented in Table 2. The LOD and LOQ values found were between 0.071 and 0.086 and 0.236 and 0.286 *μ*g mL^−1^ for Carmoisine and 0.081 and 0.091 and 0.270 and 0.304 *μ*g mL^−1^ for Ponceau 4R, respectively. The data shows that the methods are sensitive for the determination of Carmoisine and Ponceau 4R.

#### 3.6.3. Precision (Repeatability) and Accuracy (Recovery %)

The precision of the developed methods was expressed as a percentage of relative standard deviation (RSD%) for repeatability (intraday precision) and intermediate precision (interday precision). The data obtained were less than 1.06 (Table 3), indicating reasonable repeatability of the proposed methods. The intraday and interday accuracy ranges were from 98.95 to 100.6 for Carmoisine and from 99.75 to 100.7 for Ponceau 4R, respectively. The results obtained from intermediate precision (interday) also indicated a good method precision. All the data were within the acceptance criteria. To confirm the accuracy of the proposed methods, synthetic mixtures of different concentration ratios consisting of each food colorant were prepared. The resulting mixtures were assayed according to above proposed procedures in five replicates and the average results were calculated as the percentage of analyte recovered. The percentages mean accuracy for Carmoisine were 100.8 ± 0.87 for method DSM, 101.0 ± 0.95 for RDM, 100.40 ± 1.07 for DDM, and 99.75 ± 1.02 for CM, respectively. And those of P were 99.10 ± 1.02 for method DSM, 98.25 ± 0.93 for RDM, 99.55 ± 0.95 for DDM and 98.50 ± 1.06 for CM. The good recovery percent (RSD%) assured the high accuracy of the proposed methods (Table 3). The relative standard deviations were found to be less than 1.07% and 1.06% for Carmoisine and Ponceau 4R, respectively, indicating reasonable repeatability of the proposed methods.

## 4. Conclusion 

Four derivative spectrophotometric methods for the simultaneous analysis of two common food colorants have been successfully researched, developed, applied, and compared by reference HPLC method. Proposed methods provide simple, accurate, and reproducible quantitative determination of Carmosine and Ponceau 4R in synthetic mixtures and soft drinks without any interference from ingredients present. The developed methods are simple (as there is no need for pretreatment), rapid (as they require measurements of ∆D1 and D1 values at a single wavelength) and direct (as they estimate each colorant independently of the other). UV methods offer a cost effective and time saving alternative to other methods, for example, colorimetric, complexometric and chromatographic analyses. The methods used in this study are more versatile and easy to apply than the HPLC, polarographic, and voltammetric methods. Also the methods did not require any sophisticated instrumentation, such as HPLC, which requires organic solvents and time or advanced methodologies (like chemometric methods).

We conclude that the sensitivities of the proposed methods are almost comparable and can be used for the determination of the two colorants in commercial drinks in the absence of official method.

## Figures and Tables

**Table 1:** Experimental parameters calculated for the simultaneous determination of Carmoisine and Ponceau 4R in binary mixture by compensation method.

Colorants	Concentration range (µg mL^−1^)	Ratio	Mean^a^	RSD (%)
Carmoisine	2–10	^ 1^D_472_/^1^D_569_	0.600 ± 0.002	0.333
Ponceau 4R	2–10	^ 1^D_470_/^1^D_554_	0.690 ± 0.009	1.304

^a^Mean of five separate determinations.

**Table 2:** Statistical parameters of the simultaneous determination of Carmoisine and Ponceau 4R by DSM, RDM, and DDM.

Methods	Analyte	Selected wavelength (nm)	Concentration range (µg mL^−1^)	Regression equations^a^	Correlation coefficient (*r*)	Detection limit (µg mL^−1^)	Quantification limit (µg mL^−1^)
DSM	C	^ 1^D_331_	2–10	1.56 · 10^−4^ *C* + 1.90 · 10^−5^	0.9995	0.086	0.286
	P	^ 1^D_517_	2–10	2.32 · 10^−4^ *C* − 3.20 · 10^−5^	0.9996	0.091	0.304

RDM	C	^ 1^D_323_	2–10	4.13 · 10^−3^ *C* − 3.23 · 10^−4^	0.9994	0.079	0.263
	P	^ 1^D_344_	2–10	1.95 · 10^−3^ *C* − 3.43 · 10^−3^	0.9996	0.082	0.273

DDM	C	^ 1^D_366_	2–10	3.25 · 10^−4^ *C* + 1.27 · 10^−4^	0.9996	0.071	0.236
	P	^ 1^D_451_	2–10	4.39 · 10^−4^ *C* − 1.56 · 10^−4^	0.9998	0.081	0.270

^a^Five separate determinations were performed and mean was calculated.

C*: the concentration of the analyte (µg mL^−1^).

**Table 3:** Method validation for the simultaneous determination of Carmoisine and Ponceau 4R in laboratory prepared mixtures by the proposed methods.

Method		Accuracy (mean* ± RSD %)	Precision repeatability^a^ (mean ± RSD %)	Intermediate precision^b^ (mean ± RSD %)
DSM	C (^1^D_331_)	100.8 ± 0.87	100.1 ± 0.96	99.91 ± 1.02
	P (^1^D_517_)	99.1 ± 1.02	100.5 ± 1.05	100.1 ± 0.92

RDM	C (^1^D_323_)	101.0 ± 0.95	99.98 ± 0.99	100.6 ± 1.05
	P (^1^D_344_)	98.25 ± 0.93	99.83 ± 0.98	100.4 ± 0.96

DDM	C (^1^D_366_)	100.4 ± 1.07	98.95 ± 1.05	99.92 ± 0.98
	P (^1^D_451_)	99.55 ± 0.95	100.5 ± 0.95	100.7 ± 0.96

CM	C (^1^D_472_/^1^D_569_)	99.75 ± 1.02	99.8 ± 1.01	100.1 ± 0.97
	P (^1^D_470_/^1^D_554_)	98.5 ± 1.06	99.75 ± 1.03	100.3 ± 1.06

^a^The intraday (*n* = 3), average of three concentrations (2, 6, and 10 µg mL^−1^) for Carmoisine and Ponceau 4R repeated three times within the day.

^b^The interday (*n* = 3), average of three concentrations (2, 6, and 10 µg mL^−1^) for Carmoisine and Ponceau 4R repeated three times in three successive days.

*The values of % recovery are an average of five replicates of each of five synthetic mixtures at different concentration ratios of C and P (2–10 µg mL^−1^).

RSD %: relative standard deviation.

**Table 4:** Assay results for the determination of Carmoisine and Ponceau 4R in soft drinks using the proposed methods and the reference HPLC method (NMKL 130).

Methods	Analyte	Selected wavelengths (nm)	Assay results mean ± SD* (*t*_calculated_; *F*_calculated_)**	
			Soft drink I (µg 100 mL^−1^)	Soft drink II (µg 100 mL^−1^)
DSM	C	^ 1^D_331_	9.30 ± 0.22 (0.68; 1.45)	6.28 ± 0.29 (0.61; 1.44)
	P	^ 1^D_517_	2.20 ± 0.11 (1.18; 1.18)	1.39 ± 0.13 (0.40; 1.79)

RDM	C	^ 1^D_323_	9.26 ± 0.23 (0.37; 1.45)	6.36 ± 0.21 (1.28; 1.20)
	P	^ 1^D_344_	2.35 ± 0.11 (0.44; 1.25)	1.43 ± 0.13 (0.13; 1.69)

DDM	C	^ 1^D_366_	9.31 ± 0.24 (0.73; 1.69)	6.41 ± 0.24 (1.53; 1.64)
	P	^ 1^D_451_	2.49 ± 0.17 (1.89; 3.02)	1.44 ± 0.16 (0.23; 2.68)

CM	C	^ 1^D_472_/^1^D_569_	9.30 ± 0.26 (0.63; 1.88)	6.36 ± 0.28 (1.07; 2.11)
	P	^ 1^D_470_/^1^D_554_	2.30 ± 0.16 (0.23; 2.56)	1.43 ± 0.19 (0.10; 3.68)

HPLC	C	520	9.21 ± 0.18	6.20 ± 0.19
	P	512	2.32 ± 0.10	1.42 ± 0.10

*Results obtained are the average of five experiments for each method; SD: standard deviation.

**The corresponding theoretical value for *t* and *F* at *P*: 0.05 (*t*_theoretical_: 2.31; *F*_theoretical_: 6.39).
